# Withaferin A Effectively Targets Soluble Vimentin in the Glaucoma Filtration Surgical Model of Fibrosis

**DOI:** 10.1371/journal.pone.0063881

**Published:** 2013-05-08

**Authors:** Paola Bargagna-Mohan, Sunil P. Deokule, Kyle Thompson, John Wizeman, Cidambi Srinivasan, Sunil Vooturi, Uday B. Kompella, Royce Mohan

**Affiliations:** 1 Neuroscience, University of Connecticut Health Center, Farmington, Connecticut, United States of America; 2 Ophthalmology and Visual Science, University of Kentucky, Lexington, Kentucky, United States of America; 3 Statistics, University of Kentucky, Lexington, Kentucky, United States of America; 4 Pharmaceutical Sciences, University of Colorado Denver, Aurora, Colorado, United States of America; University of Regensburg, Germany

## Abstract

Withaferin A (WFA) is a natural product that binds to soluble forms of the type III intermediate filament (IF) vimentin. Currently, it is unknown under what pathophysiological contexts vimentin is druggable, as cytoskeltal vimentin-IFs are abundantly expressed. To investigate druggability of vimentin, we exploited rabbit Tenon's capsule fibroblast (RbTCF) cell cultures and the rabbit glaucoma filtration surgical (GFS) model of fibrosis. WFA potently caused G_0_/G_1_ cell cycle inhibition (IC_50_ 25 nM) in RbTCFs, downregulating ubiquitin E3 ligase skp2 and inducing p27^Kip1^ expression. Transforming growth factor (TGF)-ß-induced myofibroblast transformation caused development of cell spheroids with numerous elongated invadopodia, which WFA blocked potently by downregulating soluble vimentin and α-smooth muscle actin (SMA) expression. In the pilot proof-of-concept study using the GFS model, subconjunctival injections of a low WFA dose reduced skp2 expression in Tenon's capsule and increased p27^Kip1^ expression without significant alteration to vimentin-IFs. This treatment maintains significant nanomolar WFA concentrations in anterior segment tissues that correspond to WFA's cell cycle targeting activity. A ten-fold higher WFA dose caused potent downregulation of soluble vimentin and skp2 expression, but as found in cell cultures, no further increase in p27^Kip1^ expression was observed. Instead, this high WFA dose potently induced vimentin-IF disruption and downregulated α-SMA expression that mimicked WFA activity in TGF-ß-treated RbTCFs that blocked cell contractile activity at submicromolar concentrations. These findings illuminate that localized WFA injection to ocular tissues exerts pharmacological control over the skp2-p27^Kip1^ pathway by targeting of soluble vimentin in a model of surgical fibrosis.

## Introduction

The type III intermediate filaments (IFs) are a family of cytoskeletal proteins that display dynamic and complex expression as both soluble and polymeric proteins [1], [2], [3], [4]. Only recently, these IFs have emerged as a novel class of druggable targets [5], [6], [7], [8], [9], [10], a discovery borne out from a forward chemical genetic screening expedition investigating the binding protein targets of the anti-angiogenic natural product withaferin A (WFA) [5]. WFA was shown to bind and downregulate the soluble forms of vimentin in human vascular endothelial cells, and interestingly, this finding led further to the illumination that the WFA-binding site in tetrameric vimentin was conserved evolutionarily from humans to sharks [7]. This revelation drew our attention to WFA not only as a potent pharmacological agent but also to it's use as a chemical probe for interrogating biological signaling pathways that are linked to vimentin functions. Recognizing that vimentin, and in general the type III IFs, have roles in injury repair because of their mechanosensory functions [3], [11], we exploited a corneal alkali injury model to investigate vimentin targeting by WFA. This study demonstrated that vimentin overexpression in injured corneal tissues was downregulated by WFA resulting in potent inhibition of corneal neovascularization and scarring with consequential restoration of corneal transparency [7]. Furthermore, vimentin-deficient (Vim KO) mice were also protected against corneal fibrosis in the alkali injury model lending evidence that vimentin is the druggable target [7]. Together, these studies, along with several other findings made in other systems have illustrated vimentin involvement in epithelial mesenchymal transition [12], [13] and point to a central pathological role for vimentin overexpression in fibrotic events.

WFA exerts distinct dose-dependent inhibitory activities on cellular processes related to angiogenesis and fibrotic repair. At low nanomolar concentrations, WFA exerts G_0_/G_1_ cell cycle arrest (IC_50_ = 12 nM in endothelial cells) [14]. This cytostatic mechanism involves the expression of cyclin-dependent kinase inhibitor p27^Kip1^, which was also observed in the mouse alkali-injury model of corneal fibrosis when animals were treated with WFA [7]. The perturbation of the cell cycle results from WFA's downregulation of the ubiquitin E3 ligase, skp2. This downregulation alleviates the proteasome-mediated degradation of p27^Kip1^, which is a target of skp2, and promotes corneal cell cycle arrest. That this skp2-p27^Kip1^ pathway is critical to WFA's cell cycle-targeting mechanism was illustrated in embryonic fibroblasts derived from either skp2-deficient or p27^Kip1^-deficient mice that showed resistance to cell cycle blockade after treatement with WFA when compared to wild-type cells [7]. At higher concentrations in the submicromolar range, WFA also inhibits cell migration and cell invasion (IC_50_ 500 nM), which are critical for endothelial cell sprouting in three-dimensional collagen gel spheroid angiogenic assays [14], [15], [16]. Over this higher submicromolar concentration range, WFA also perturbs the vimentin-IF cytoskeleton causing the polymeric forms to condense at the perinuclear region with ensuing cell shape changes as a result of cytoskeletal retraction [5], [17], [18]. Above 2 µM WFA induces extensive perturbation of the IF-cytoskeleton, fragmentation of F-actin and induction of apoptosis, which is a critical mechanism for cancer chemotherapeutics [8], [9]. As such, the low nanomolar effects of WFA on soluble IFs are not detectable by immunohistochemical methods because soluble IFs represent less than 5–10% of total IF protein pool in most primary cell cultures. However, growth-arrested cells stimulated to enter the cell cycle have a significantly larger pool of tetrameric vimentin that rapidly undergo polymerization into filamentous forms upon cell cycle entry; it is at this cell cycle stage that WFA exerts its most potent inhibitory activity on cell proliferation [6], [7], [14]. Appreciating that such dynamic states for soluble and polymeric vimentin have also been reported in different biological contexts [2], [19], [20], [21], [22], [23], , the vulnerability of soluble vimentin [1] in living tissues to WFA's pharmacological activity has remained poorly characterized.

The importance of vimentin expression is especially relevant to wound repair [27], [28]. Vim KO mice display delayed wound healing when subjected to full thickness skin injury, but these mice are otherwise normal and fertile. This phenotype is characterized by a delay in myofibroblast influx at the wound site, which results from deficiencies in cell migration and contractile forces necessary to seal an open wound [28]. Whereas complete absence of vimentin may not be desirable for wound repair in vascularized tissues, the benefit of reduced myofibroblast involvement and attenuated cell proliferation promoted by pharmacological downregulation of vimentin offers therapeutic advantages because conditional control of target expression can be pharmacodynamically modulated [7]. However, to investigate vimentin druggability in ocular models of injury repair, a localized delivery method to evaluate WFA activity *in vivo* would be more clinically relevant, because this strategy also avoids systemic effects [29].

Glaucoma filtration surgery (GFS) is a procedure used to alleviate increased intraocular pressure in patients who have high-tension glaucoma [30]. The procedure involves creating an alternative passage to bypass blocked or failing trabecular meshwork. The success of this surgical procedure is compromised when excessive cell proliferation in Tenon's capsule by fibroblasts causes the surgical bleb to develop scar tissue [31]. A key pro-fibrogenic factor found elevated during glaucoma wound repair is the cytokine transforming growth factor (TGF)-β [31], [32]. Exposure to TGF-β expression after GFS can cause scarring by recruiting Tenon's capsule fibroblasts to differentiate into myofibroblasts and exert contractile activity [33], [34]. Treatment of human trabecular meshwork cells with TGF-β1 or TGF-β2 promotes strong overexpression of vimentin [33]. Vimentin overexpression has been implicated in the GFS model of fibrosis; vimentin transcripts were found increased in Tenon's capsule over the temporal development of scarring in rats [35]. Moreover, skp2 overexpression in the rabbit GFS model of fibrosis has also been demonstrated and its downregulation by siRNA therapy rescues Tenon's capsule from bleb failure [36]. Furthermore, gene therapy to increase abundance of p27^Kip1^ expression in the GFS rabbit model has also shown protection against bleb failure [37]. Taken together, the skp2-p27^Kip1^ pathway is likely a critical target for anti-fibrotic modalities in GFS fibrosis, but whether vimentin targeting affects this key cell proliferation signaling pathway in GFS has remained untested.

The objective of this study was to identify a dose of WFA for localized delivery that was effective at targeting soluble vimentin *in vivo* and producing significant effects on the skp2-p27^Kip1^ pathway. Towards this goal, we exploited the RbTCF cell culture model [38] to first determine the concentration-dependent effects of WFA on cell proliferation, differentiation and migration, and interrogate WFA's effects on the skp2-p27^Kip1^ pathway [7]. Our *in vivo* proof-of-concept study used the rabbit GFS preclinical model of surgical fibrosis [39]. Two doses of WFA differing by 10-fold concentration were tested by localized delivery to differentially target soluble vimentin over cytoskeletal vimentin-IFs. Here, we report that fibrotic biomarker (skp2, p27^Kip1^ and α-SMA) expression was differentially altered in manners similar to the WFA dose-response found in cell cultures.

## Methods

### Reagents and general methods

WFA was purchased from Chromadex (Santa Ana, CA, USA). Cell culture supplies were purchased from Life Technologies (Grand Island, NY, USA) and cell culture plates from Thermo Fisher Scientific (Pittsburgh, PA, USA), unless otherwise specified. Mitomycin-C (MMC), and dimethylsulfoxide (DMSO) were purchased from Sigma-Aldrich (Saint Louise, MO, USA).

### Isolation and culture of Rabbit Tenon Capsule Fibroblasts (RbTCFs)

To isolate RbTCFs, we followed established protocols with minor modifications [40] [41]. Briefly, fresh young rabbit eyes shipped overnight after sacrifice (Pel-Freez Biologicals, Rogers, AR, USA), upon arrival, were washed three times with saline solution containing an antibiotic-antimycotic cocktail and subconjunctival Tenon's capsule tissues (10–15 mm^2^ approximate area of each sample; n = 26 total) were dissected and transferred, in sterile condition, to 60 mm culture plates. Each tissue was gently pressed against the bottom of the plate and kept attached by placing a sterile round glass coverslip on top of the tissue. Tissues were overlaid with DMEM/F12 medium containing 10% fetal bovine serum (FBS), L-glutamine and an antibiotic-antimycotic cocktail (DMEM complete medium). Cultures were incubated at 37°C in a humidified chamber maintained under 5% CO_2_ and cell culture medium was replaced every 2 days. After 14 days in culture, when numerous RbTCF cells had migrated out from the explants the tissue pieces were carefully removed. RbTCF cells were allowed to reach confluency and then sub-cultured for the designed experiments. Cells were used before 6^th^ passage.

### WFA treatment

RbTCF cells were plated at 50% confluence in 100 mm plates in complete DMEM/F12 medium and allowed to attach for 4 h. Cells were serum-starved in DMEM/F12 medium containing 0.1% FBS for 48 h and then treated with different doses of WFA in the presence of serum (10% FBS) for 24 h. Soluble and insoluble protein fractions were extracted for western blot analysis as previously described [6]. For immunohistochemistry analysis, cells were seeded into 8 well-glass slides in the presence or absence of different concentrations of WFA for 24 h. Cells were fixed and processed for fluorescence staining as described previously [6].

### Cell culture scratch wound assay

RbTCF cells were seeded on 8-well-glass slides at 95% confluency in DMEM/F12 complete medium and cells were incubated for 24 h to allow to uniform covering of the glass well. A cross-stripe scrape was made in each well, by using a 100 µl micropipette tip and the floating cells and debris were gently washed away. Adherent cells were incubated for 24 h in the presence or absence of different concentrations of WFA or MMC (15 µM), used as positive control. Cell migration was stopped at 24 h post-injury, when the control samples showed 95% closure of the wound. Photographs were taken at 4× magnification using an inverted microscope (model CKX 41, Olympus America Inc., Center Valley, PA, USA) equipped with a digital camera (Q Color3) at the time the scrape wound was introduced and 24 h post-wounding. Images (n = 3/each treatment) were imported to NIH ImageJ software and the empty cell-free gap of each wound (perpendicular to the axis of injury) was measured at eight separate spots at time 0 h, using as measurement unit the “scale bar” tool in the ImageJ program. After 24 h, the thickness of the gap left remaining between the two migratory edges of each wound was measured again (n = 8). For each treated and control group, we obtained 24 arbitrary values from which we calculated the average and standard deviation using Microsoft Excel. To measure the extent of cell proliferation contributing to wound closure, cell cultures from 24 h post-injury samples were additionally subjected to staining for Ki67 and counter stained with DAPI (as described in Immunohistochemistry analysis section). DAPI-stained nuclei of cells lining the wound margin as well as those that were within the wound were counted in 6 independent fields for each treatment condition.

### Cell cycle analysis

For cell cycle analysis we followed a previously published protocol (19) with some minor modifications. Briefly, RbTCF cells were grown to confluence in 100 mm plates and growth-arrested by serum starvation for 48 h in DMEM/F12 medium containing 0.1% FBS. Cells were trypsinized and seeded into 100 mm culture plates at 60% confluency and allowed to attach for 2 h. Cells were treated with vehicle (DMSO) or different concentrations of WFA in the presence of serum (10% FBS) for 24 h and 72 h. Cells were stained with 50 mg/ml propidium iodide and processed for flow cytometric analysis following manufacturer's protocol

### Gel contraction assay

Collagen type I was extracted from rat tails [42] and formulated as previously described [14]. Briefly, the ice-cold clear collagen stock solution was neutralized with 0.2N NaOH, 50 mg/ml NaHCO_3_ and 1∶1000 diluted solution of acetic acid in 10× DMEM/F12 medium. This solution was immediately mixed with RbTCFs at a concentration of 2×10^5^ cells/ml and 100 µl of the collagen solution-cell mix was plated into a 96-well tissue culture plate (n = 6 gels per treatment group). Gels were allowed to polymerize in an incubator at 37°C with 5% CO_2_ for 30 min. Polymerized gels were gently detached from the well sides and different concentrations of WFA were added and gels cultured in the presence or absence of recombinant human TGF-ß1 (2 ng/ml; R&D Systems, Minneapolis, MN) well for 3 days. Gel contractile activity was visualized at 10× magnification with the aid of an inverted microscope, and digital images were imported to image-management software (Photoshop; Adobe Systems, Mountain View, CA) for evaluation and analysis.

### *In vitro* myofibroblast transformation

RbTCFs were grown to confluence in 60 mm plates and growth-arrested by serum starvation for 48 h in DMEM/F12 medium containing 0.1% FBS. After cell cycle arrest, cells were trypsinized and replated in presence of serum (10%) in 100 mm plates at 60% confluency. For western blot analysis, cells were seeded in 100 mm plates at 60% confluence and for immunohistochemistry analysis cells were seeded into 8-well glass slides. In both sets of experiments, cells were allowed to attach for 3 h prior to treatments. Cells were washed once with 1× PBS to remove debris and floating cells. Cells were then treated with different concentrations of WFA and cultured in the presence or absence of TGF-ß1 (2 ng/ml) for 3 days. After 72 h treatment, cells were prepared for western blot and immunohistochemical analysis as described in the following sections.

### Glaucoma filtration surgery in rabbit and WFA treatment

The animal experiments were conducted in strict accordance with the Declaration of Helsinki, and under approved protocol (Protocol number 2010-0682) by the Institutional Animal Care and Use Committee of the University of Kentucky. All surgeries were performed by the glaucoma clinical specialist (S.D.) who has expertise in the rabbit model of glaucoma filtration surgery (GFS). Anesthesia was employed during surgery and all efforts were made to minimize animal suffering. New Zealand white male rabbits weighing 2.5 to 3.0 kilogram each were employed in the study. All animals were housed in separate cages under a 12 h light dark cycle with food and water available *ad libitum* and showed normal intraocular pressure (IOP) before the experimental procedure. GFS was performed on both eyes: right eyes (n = 6) received 100 µl-WFA injections and left eyes (n = 6) received 100 µl-saline injections (vehicle-treated samples). Rabbits were anesthetized with ketamine-xylazine (25 mg/kg and 2 mg/kg, respectively). A superior conjunctival flap was then created and a 22-gauge cannula was inserted in the anterior chamber. The cannula was secured with sclera using 8'0 prolene suture and conjunctival flap was sutured using 8'0 vicryl. The cannula drains aqueous from the anterior chamber under the conjunctiva thus creating filtering bleb. Antibiotic and steroid eye ointment was placed in each eye at the end of surgery. To investigate WFA's targeting of soluble vimentin in the GFS model, we compared the effects of two doses (0.7 µg and 7.0 µg) that were directly injected under the conjunctiva 4–5 mm above the filtering bleb. The WFA drug formulations were freshly prepared as an emulsion in sterile saline each day prior to injection, which was delivered above the conjunctival bleb. Injections of WFA or vehicle were provided at the end of the surgery (day 0) and subsequently, on days 4, 7, 14 and 21 after local topical application of tetracaine 1% eye drops. Clinical eye examination, including assessment of bleb height, bleb vascularity, corneal clarity and anterior chamber inflammatory reaction, was conducted at d7, d14 and d21 post-surgery to obtain useful information with respect to potential future drug efficacy studies. Intraocular pressure was recorded at each time point under topical anesthesia using Tonopen-Vet (Reichert Inc., Depew, NY, USA). Bleb survival or failure was recorded at each time point. Bleb was considered as failed if it was found to be flat, highly vascularized or lacking any vascularization. Animals were sacrificed on d28, whole eye globes enucleated and frozen at −80 °C until use. Tenon's capsule biopsies were dissected from uninjured (Control) eyes, and from GFS-injured eyes and tissues prepared for western blot and immunohistochemical analyses.

### Immunohistochemistry analysis

For immunostaining of cell cultures, cells were fixed in ice-cold methanol for 5 min and then in acetone for 1 min at −20°C. After a brief wash, cells were permeabilized/blocked in a 0.1% Triton X-100 solution containing 1% albumin and 5% normal goat serum for 1 h at 37°C. According to individual experiments, cells were labeled with either rabbit anti-Ki-67 antibody (1∶200, Santa Cruz Biotechnology, Inc., Santa Cruz, CA, USA) or rabbit anti-vimentin antibody (1∶100, Abcam, Cambridge, MA, USA) for 18 h at 4°C in a background reducing solution (Dako North America Inc., Carpinteria, CA, USA). Cells were then incubated with goat anti-rabbit IgG secondary antibodies conjugated to Alexa Fluor 488 (1∶1000, Life Technologies, Grand Island, NY, USA) at room temperature for 45 min. Nuclei were stained with 4,6-diamidino-2-phenylindole (DAPI, 1 mg/mL in 0.1 M PBS) for 10 min [6]. In some experiments, cells were also counterstained with Phalloidin (1∶500, Life Technologies, Grand Island, NY, USA) for 1 h at room temperature. Digital images were acquired on an Olympus IX81 microscope at 4× and 30× magnification and were digitally deconvolved using AutoQuant Deconvolution software (Olympus America Inc., Center Valley, PA, USA) to improve image contrast and resolution [7]. Images were assembled using Adobe Photoshop Software.

For immunostaining of rabbit tissues, samples were fixed in 4% PFA, and then permeabilized/blocked in a 0.1% Triton X-100 solution containing 1% albumin and 5% normal goat serum for 1 h at 37°C. Samples were stained with mouse anti-vimentin antibody (clone V9, 1∶100, Santa Cruz Biotechnology, Inc., Santa Cruz, CA, USA) for 18 h at 4°C in a background reducing solution (Dako North America Inc., Carpinteria, CA, USA). After a brief wash, slides were incubated with goat anti-mouse IgG secondary antibodies conjugated to Alexa Fluor 555 (1∶500, Life Technologies, Grand Island, NY, USA) for 45 min at room temperature and counterstained with DAPI (1 mg/mL in 0.1 M PBS) for 10 min. Digital images were acquired on an Olympus IX81 microscope at 4× and 30× magnification and were digitally deconvolved using AutoQuant Deconvolution software to improve image contrast and resolution [7]. Images were assembled using Adobe Photoshop Software.

### Western blot analysis

For cell culture analysis, insoluble and soluble proteins were extracted as previously described [6], [7]. Equal amounts of protein were subjected to SDS-PAGE on 4–20% gradient polyacrylamide gels (Biorad, Hercules, CA, USA) and transferred to a PVDF membrane. Blots were probed with mouse anti-vimentin antibody (clone V9, 1∶400, Santa Cruz Biotechnology), mouse anti-skp2 antibody (clone A-2, 1∶200, Santa Cruz Biotechnology Inc., Santa Cruz, CA, USA), rabbit anti-p27^kip1^ antibody (1∶200, Santa Cruz Biotechnology Inc., Santa Cruz, CA, USA), and mouse anti-ß-Actin antibody (clone AC-15, 1∶20,000, Sigma-Aldrich, St. Louis, MO, USA). After extensive washing and incubation with respective secondary antibody conjugated to horseradish peroxidase, the membrane was developed using chemiluminescent reagents (Amersham, Piscataway, NJ, USA) and exposed to X-ray films. Blots were scanned and band intensities quantified using NIH ImageJ software and normalized to ß-actin levels.

For protein analysis from Tenon's capsule tissues, soluble proteins from each sample were extracted using methods previously described [6]. Equal amounts of protein were subjected to SDS-PAGE on 4–20% gradient polyacrylamide gels (Biorad, Hercules, CA, USA) and transferred to PVDF membranes. Semi-quantitative analysis by western blotting was performed and blots were probed with mouse anti-α-SMA antibody (1∶200, Dako), mouse anti-vimentin antibody (clone V9, 1∶400, Santa Cruz Biotechnology, Santa Cruz, CA, USA), mouse anti-skp2 antibody (1∶200, Santa Cruz Biotechnology Inc., Santa Cruz, CA), rabbit anti-p27^kip1^ antibody (1∶200, Santa Cruz Biotechnology Inc., Santa Cruz, CA) and mouse anti-GAPDH antibody (1∶1000, Abcam, Cambridge, MA, USA). Blots were scanned and band intensities quantified using NIH ImageJ software and normalized to GAPDH levels.

### Statistical analysis

The target validation proof-of-concept studies employed a statistical evaluation scheme for pharmacodynamic (PD) measurements of soluble vimentin expression as the end point, which was made by western blot analysis. A significant change was defined by expression levels of soluble vimentin attained 28 days after injury that were increased by greater than 50% and that this change was sufficient when compared to the baseline variation of this protein's levels in uninjured rabbit Tenon's capsule. With these two criteria, a 95% statistical confidence that the measured differences were not due to chance variation was established. In addition, to establish that drug-induced effects also accounted for biomarker changes in Tenon's capsule, we performed simultaneous comparisons with skp2, p27^kip1^ and α-SMA and defined the results to be significant at P<0.05 using one-sided tests. Pearson correlations on the log-transformed values were conducted to determine relationships between proteins in injured tissue and WFA-treated samples. The inverse relationship was used for p27^kip1^ because of the known mechanism of skp2-dependent p27^kip1^ downregulation in ocular injury repair [43].

### Analysis of withaferin by liquid chromatography and tandem mass spectrometry

Since Tenon's capsule was employed for analysis of protein biomarkers, we used remaining tissues of the anterior segment such as the cornea, aqueous humor, ciliary body-iris and conjunctiva [44] to estimate WFA tissue concentrations in close proximity to the injection site. Tissue samples were incubated in 250 µl of water containing the internal standard (500 ng/ml), and vortexed for 10 min. Subsequently, tissue samples were homogenized using a hand-held homogenizer (Tissue Tearor, Biospec Products, Bartlesville, IL, USA). To this tissue homogenate, 750 µl of ethyl acetate was added and vortexed for 20 min. Tissue homogenates were centrifuged at 13,000×g for 10 min to separate out the precipitated proteins. The supernatant was removed and 1 ml of fresh ethyl acetate was added to the tubes and vortexed for 20 min. Tubes were centrifuged again, and both supernatants were transferred to glass tubes and the solvent was evaporated using a nitrogen evaporator (Multivap, Organomotion, Berlin, MA, USA) for 40 min. The residue was reconstituted in 0.25 ml of acetonitrile and water (1∶1) and transferred into LC-MS vials and subjected for analysis. Prednisolone acetate was used as an internal standard. WFA and prednisolone acetate were extracted from rabbit ocular tissue (100 mg) by the ethyl acetate based protein precipitation method [45]. The HPLC run conditions employed 10% acetonitrile-water gradient starting from 0 to 0.5 min, followed by an increasing acetonitrile gradient (10 to 90%) over the next 6 min followed by a decreasing acetonitrile gradient (90 to 10%) at 8 min till the run completion at 9 min. Calibration curve samples were prepared using choroid retinal pigment epithelium (CRPE) tissue from untreated rabbit eyes obtained from Pel-Freez Biologicals (Rogers, AR, USA). Extraction recovery of WFA from these blanks was estimated at three different (1, 10, and 100 ng/ml) concentrations using CRPE tissue.

WFA concentrations in extracts from rabbit ocular tissue samples were measured by means of electrospray ionization MS/MS analysis using an API-4000 triple quadrupole mass spectrometry (Applied Biosystems, Foster City, CA, USA) following separation by HPLC. Analytes were separated on a Zorbax C18, 4.1×50 mm, 5 µm column using acetonitrile and 5 mM ammonium formate in water (adjusted to pH 3.5 with formic acid) as mobile phase in gradient elution mode with a flow rate of 0.40 ml/min. The total run time of analysis was 9.0 min. Both WFA and prednisolone acetate were analyzed in positive ionization mode.

## Results

### WFA targets soluble vimentin and inhibits cell cycle progression in RbTCFs mediated by the skp2-p27^kip1^ pathway

**Figure 1:**
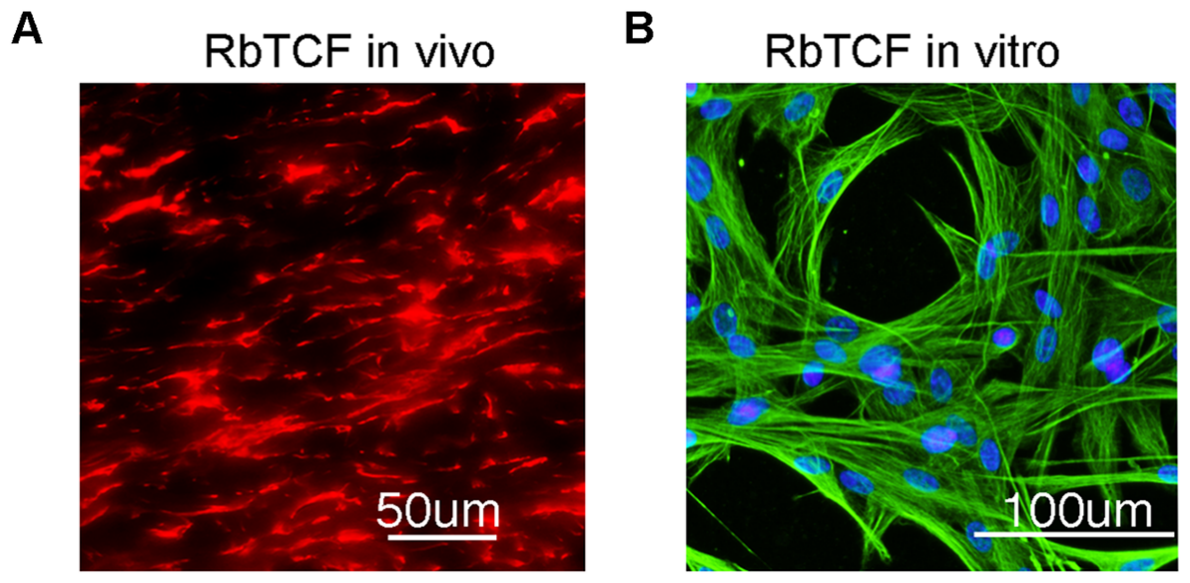
Identification of RbTCF cells *in vivo* and *in vitro*. Immunohistochemistry analysis of cell marker vimentin in rabbit Tenon's capsule tissue (B, *red*) and in isolated RbTCFs (A, *green*). Scale bars: 100 µm *in vitro* (A), 50 µm *in vivo* (B).

**Figure 2:**
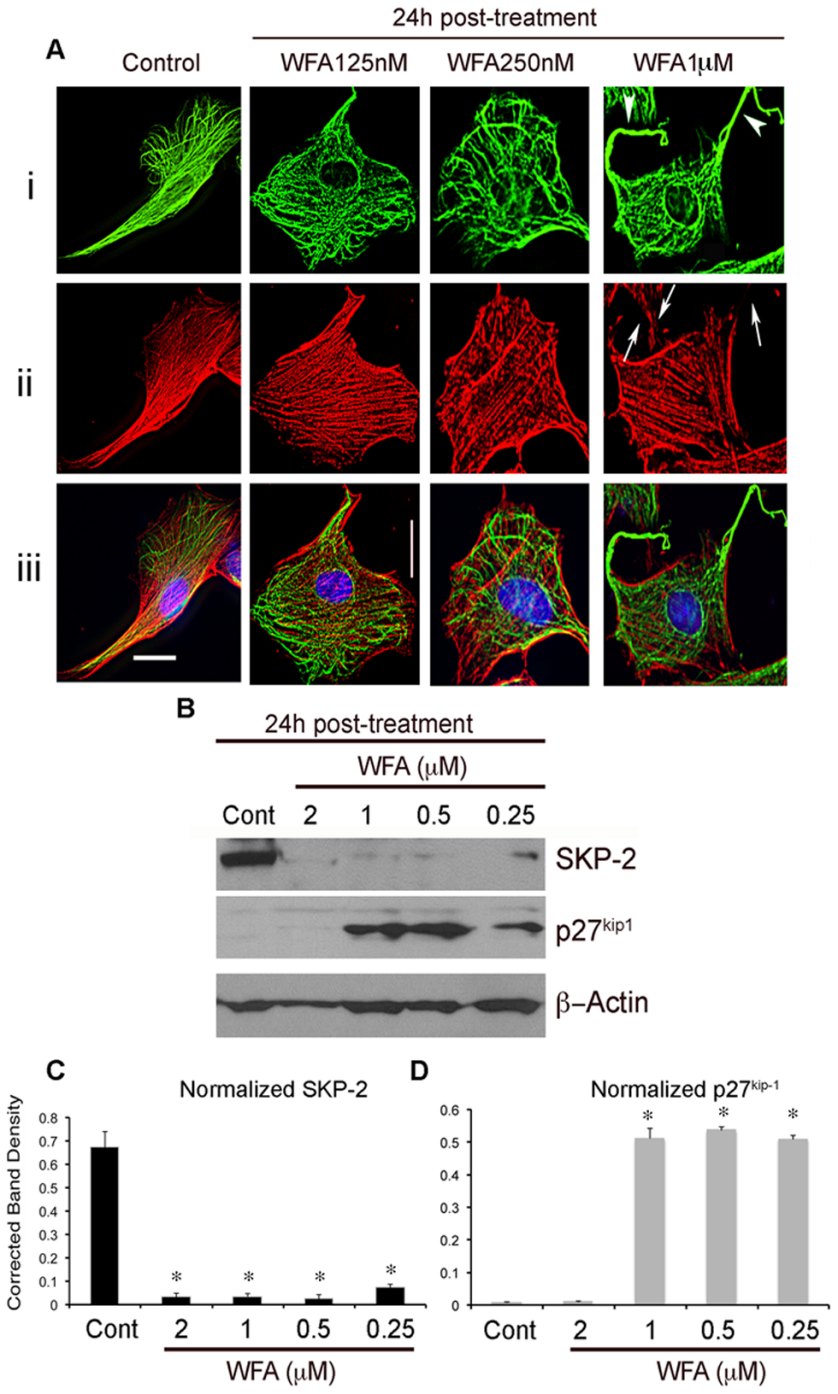
WFA's cell cycle effects are regulated by the skp2-p27^kip1^ pathway in RbTCF cells. (A) Representative images of RbTCFs stimulated to enter the cell cycle after 48 h serum starvation. Cells were stained with vimentin (*green*) and phalloidin (*red*) at 24 h post-WFA treatment. WFA's low concentration cell cycle activity does not affect the vimentin-IF network (*green*). Vimentin-IFs become affected only at higher concentrations (1 µM, *arrow heads*) and leads to dismantling of the actin fibers (*red*) that are observed at the periphery of cytoplasm (*arrows*). (B) Western blot analysis of skp2 and p27^kip1^ expression in RbTCF cells at 24 h post-WFA treatment. C) Densitometric quantification of skp2 (*black*) and p27^kip1^ (*grey*) normalized to β-actin, using ImageJ software. Scale bars: 30 µm at 30× magnification. * P<0.05 WFA vs control.

Vimentin staining has been employed as a marker for Tenon's capsule fibroblasts [40], which is illustrated here in tissue sections showing the abundant expression of this cytoskeletal protein (Figure 1A). Primary cell cultures established from Tenon's capsule maintain a cytoskeletal vimentin expression pattern that is typical for fibroblasts (Figure 1B). Next, to identify concentration-related effects of WFA that cause perturbation of the vimentin-IF cytoskeleton we investigated RbTCFs by immunostaining. At higher concentrations (1 µM), WFA caused severe re-arrangement of the vimentin network causing the peripheral vimentin-IFs to collapse (Figure 2A–i) and condense around the nucleus. At low concentrations (0 to 500 nM), WFA did not affect vimentin-IFs as evidenced by their staining (Figure 2A–i, *green*). Since we had previously shown in other cell types [5], [6] that such higher concentrations of WFA also interfere with F-actin, thought to occur through vimentin-mediated association with filament-binding partners [5], [46], we performed staining with phalloidin to examine F-actin. F-actin staining also remained intact at the low concentrations of WFA (Figure 2A–ii, *red*), whereas, at the higher WFA concentration of 1 µM, F-actin collapse was observed primarily at the distal ends of the cytoplasm (Figure 2A–ii, *arrows*), where the retraction of vimentin-IFs was prominent (Figure 2A–i, *arrowheads*). Next, to determine WFA's inhibitory activity on cell cycle progression [6], [14] we investigated the cytostatic mechanism of WFA in RbTCFs. Growth-arrested RbTCFs induced to proliferate in response to serum in the presence of WFA showed distinct G_0_/G_1_ dose-related cell cycle inhibition (IC_50_ = 25 nM) (Table 1). Previously, we had identified that the cyclin-dependent kinase inhibitor p27^kip1^ is critical to WFA's inhibition of cell proliferation [7]; therefore, we also investigated the expression of p27^kip1^ in RbTCFs by western blot analysis (Figure 2B). Proliferating vehicle-treated cells expressed very low levels of p27^kip1^, whereas, WFA treatment increased p27^kip1^ expression that plateaued around 250 nM and reached maximal expression at 500 nM (Figure 2D). At 2 µM, WFA caused p27^kip1^ levels to decrease strongly, which was associated with severe vimentin-IF cytoskeletal perturbation and F-actin disintegration followed by apoptosis (data not shown). Because of its critical role in the cell cycle regulation of p27^kip1^ expression, we investigated the expression of ubiquitin E3 ligase skp2 [47]. Skp2 expression levels in vehicle-treated cells were clearly high (Figure 2B), as it is known that skp2 expression becomes induced at G_1_ and remains high through S/G_2_ [48]. Remarkably, WFA treatment at 250 nM caused skp2 expression to be significantly downregulated and to remain downregulated over the WFA-treatment dose curve (Figure 2C). Together, these data show that WFA potently interferes with the skp2-p27^kip1^ pathway to maintain inhibitory control over the cell cycle at low concentrations.

**Table 1:** WFA potently arrests RbTCFs in G_0_/G_1_ phase.

Treatments	S (%)	G_2_/M (%)	G_0_/G_1_ (%)
Control 0 h	0.00	6.97	93.03
Control 24 h	22.36	21.39	56.26
WFA 30 nM	8.01	13.18	78.81
WFA 62 nM	6.65	9.70	83.66
WFA 125 nM	2.17	2.64	95.19
WFA 500 nM	0.10	2.10	97.88
WFA 1 µM	2.24	1.60	96.16

# mean of two experiments.

### WFA downregulates soluble vimentin and attenuates TGF-β-induced myofibroblast differentiation in RbTCF cells

**Figure 3:**
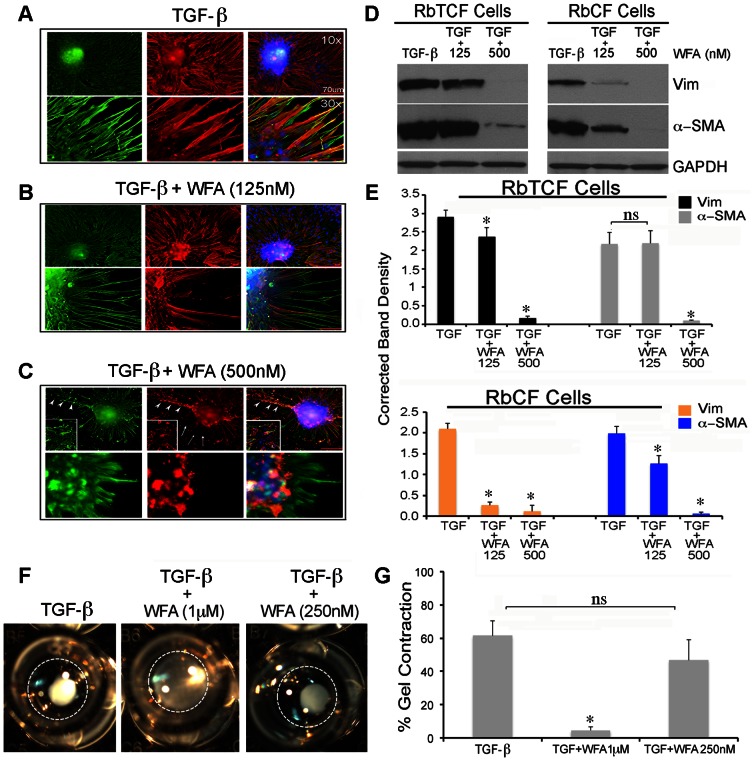
WFA prevents myofibroblast transformation and collagen gel contraction in RbTCF cells. Immunohistochemical analysis of vimentin (*green*) and α-SMA (*red*) in RbTCF cells treated with TGF-β1 (2 ng/ml) for 3 days in presence and absence of WFA. (A) TGF-β1 treatment induces formation of spheroid-like agglomerates (upper panels, 10× magnification) with sprouting extensions positive for vimentin (*green*) and α-SMA (*red*) (lower panels, 30× magnification). (B–C) WFA causes a dose-dependent downregulation of both vimentin (*green*) and α-SMA (*red*) expression. Upon treatment with 500 nM WFA, cells form spheroids poorly (C, insert panels) and those that do form spheroids have shorter and thinner extensions (C, *arrowheads*). Scale bars: 70 µm at 10× magnification; 30 µm at 30× magnification. (D) Western blot analysis of soluble vimentin and α-SMA expression in RbTCF cells (left panel) and RbCF cells (right panel). (E) Densitometric quantification of vimentin (*black and orange*) and α-SMA (*grey and blue*) in RbTCF cells and RbCF cells, respectively, normalized to GAPDH using ImageJ software. (F) Representative images of polymerized collagen gels containing RbTCF cells (n = 6 gels per treatment group) treated with TGF-β1 (2 ng/ml) in presence and absence of different doses of WFA for 3 days (10× magnification). (G) Quantification of percentage of gel contraction normalized to the initial size of the gel. Data are the mean of 2 independent experiments; Dotted circles represent the well's area. *P<0.05 TGF-β vs WFA treated cells.

Next we investigated the concentration-dependent activity of WFA in RbTCFs on TGF-β-induced myofibroblast transformation. Interestingly, RbTCFs exposed to TGF-β1 (2 ng/ml) for 3 days induced the formation of spheroid-like cell agglomerates with remarkable orchestration of sprouting filopodia that stained positive for both vimentin (*green*) and α-SMA (*red*) (Figure 3A). This phenotype revealed the successful transformation of RbTCFs into myofibroblasts with a gain of invasive characteristics [13]. RbTCFs treated with TGF-β1 (2 ng/ml) in the presence of 500 nM WFA caused not only the fragmentation of filamentous vimentin (Figure 3 C, higher inset panel, *green*, *arrowheads*), but filopodia also had shorter protrusions (*arrowheads)* due to considerable cytoskeletal effects on both vimentin-IFs (*green*, *arrowheads*) and α-SMA (*red*, *arrows*) expression. To clarify whether the perturbation of vimentin-IFs was due to targeting of soluble vimentin by WFA, we also employed western blotting to analyze the cellular soluble protein fractions. As seen in Figure 3D, WFA's targeting of soluble vimentin caused its downregulation that was clearly evident at 125 nM, whereas, α-SMA expression remained unperturbed. However, at 500 nM WFA, soluble vimentin was completely downregulated, and α-SMA expression also showed a 10-fold reduction (Figure 3D). We previously had reported that WFA downregulates α-SMA expression in rabbit corneal fibroblasts (RbCFs) when cultured in presence of TGF-ß1 [7], and therefore, we compared these two types of ocular fibroblasts in parallel. We found that RbCFs were about 6- to 8-times more sensitive to WFA compared to RbTCFs when cultured in the presence of TGF-ß1 (Figure 3D). Notably, corneal fibroblasts did not form spheroid cell agglomerates when cultured in the presence of TGF-ß1, although their competency to differentiate into myofibroblasts remained unperturbed [7]. Finally, to test whether WFA interfered with TGF-ß-induced contractile function in RbTCFs, we also employed a collagen gel contraction assay using RbTCFs (Figure 3F). We found that RbTCFs in presence of TGF-ß1 contract collagen gels. WFA co-treatment at low concentrations (250 nM) did not affect the contractile activity of RbTCF cells, but significant attenuation of collagen gel contraction (over 80% reduction) occurred when the concentration of WFA was increased to 1 µM (Figure 3F). Together these data demonstrate that soluble vimentin was more effectively targeted by WFA in corneal fibroblasts in comparison to RbTCFs in presence of TGF-ß1. Despite these cell type-sensitivity differences, RbTCFs acquire greater sensitivity to WFA in the presence of TGF-ß1 compared to serum treatment.

### WFA potently downregulates cell migration

**Figure 4:**
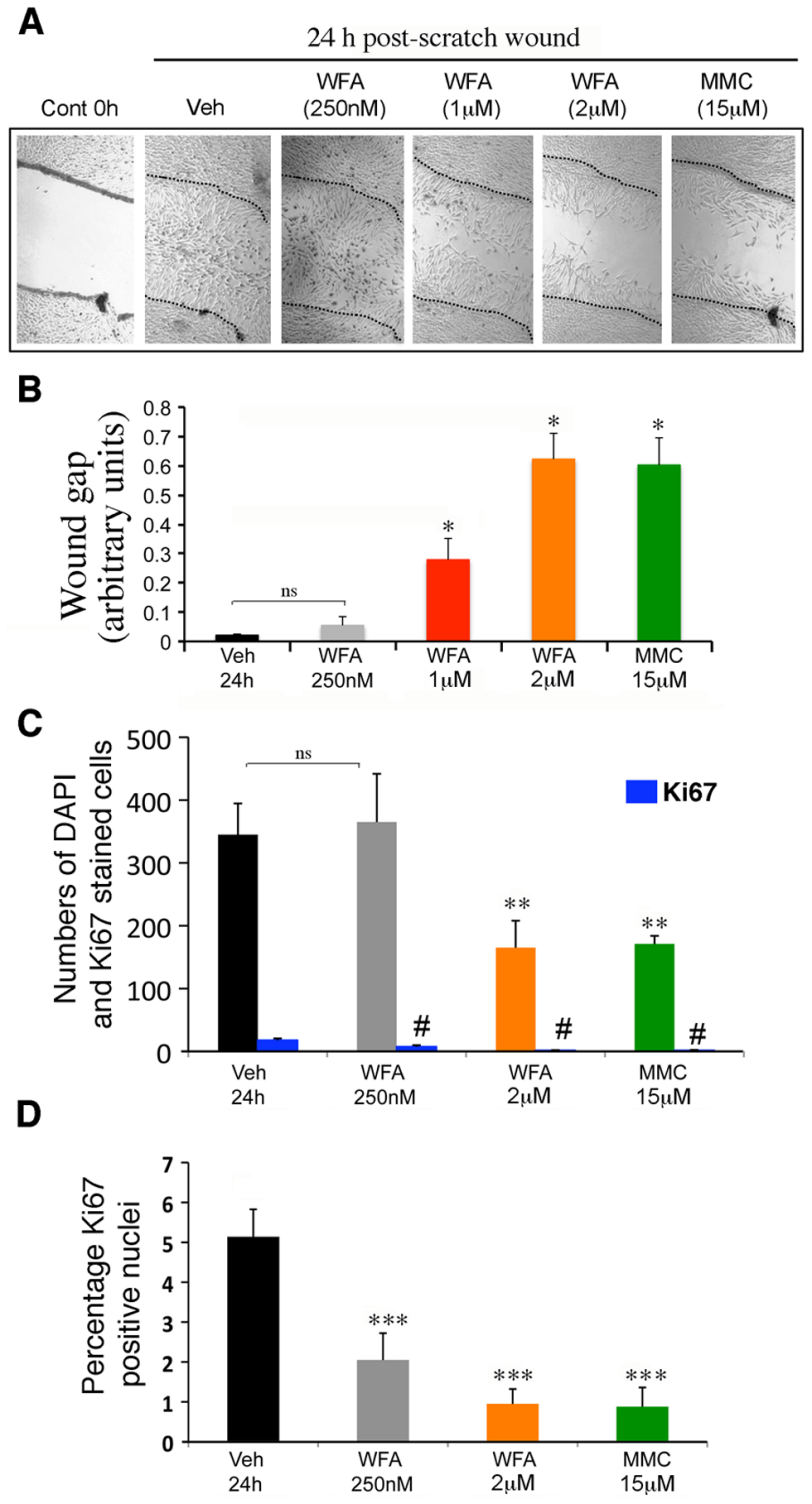
WFA inhibits cell migration in RbTCF cells. Scratch wound assay *in vitro*. (A) Representative phase-contrast images of RbTCF cells immediately after a cross-stripe scrape injury on a confluent cell layer (0 h), 24 h post-wounding (Veh), and after treatment with different concentrations of WFA. Mitomycin-C (MMC; 15 µM) was used as positive control. (B) Arbitrary values of wound gap at 24 h normalized to size of initial wound gap and calculated from two independent experiments (n = 3 for each treatment group). *P<0.05 vehicle vs drug treated; ns = non-significant. (C) Quantification of total numbers of DAPI nuclei per wound area at 24 h post-scratch injury (**P<0.0001 vehicle vs drug treated) and Ki-67 positive cells (blue bars; ^#^P<0.0001 vehicle vs drug treated). (D) Fraction of Ki67 positive nuclei in the wound area as a percentage of DAPI nuclei at 24 h post-scratch injury. *** P<0.0001 vehicle vs drug treated.

WFA is also known to inhibit cell migration [9], [14] and a number of studies have reported this activity occurs at concentrations that are not toxic to cells [8], [9], [18]. Since cell migration is an obligatory step during a wound healing response, we investigated RbTCF cell migration in a culture model of wound closure to determine WFA's dose-response in this system. As shown in Figure 4A, untreated RbTCFs were able to cover 95% of the surface area left exposed from the scratch wound within 24 h. In the presence of 250 nM WFA RbTCF cell migration into the wound area was unaffected. However, significant inhibition of cell migration occurred in samples treated with 1 µM and 2 µM WFA (P<0.001). Importantly, even at the highest dose tested (2 µM) we did not observe WFA-induced toxicities, which was evident from absence of cell rounding and loss of cell attachment. In comparison, MMC achieved similar levels of inhibition in the scratch wound assay at concentrations 7-fold higher than that of WFA (Figure 4B). Next, to ascertain that wound closure primarily reflected cell migration we also stained RbTCFs for Ki67 to examine the numbers of proliferating cells present in the wound area (see Methods). As compared to vehicle treatment, 250 nM WFA did not affect the numbers of DAPI positive nuclei found in the wound area (P = 0.595). However, as anticipated, the 2 µM WFA and 15 µM MMC treatments caused significant reduction (2-fold; P<0.0001) in numbers of DAPI-positive cells in the wound area. Remarkably, co-labeling for Ki67-positive nuclei in the vehicle-treated condition revealed that only 5% of the DAPI nuclei present in the wound area expressed this proliferation marker (Figure 4C). WFA treatment caused a dose-dependent decrease in numbers of these Ki67-positive cells to 2% at 250 nM (P<0.0001) and to 1% at 2 µM concentration (P<0.0001) (Figure 4D). MMC had a similar effect as 2 µM WFA on numbers of Ki67-positively stained nuclei (P<0.0001). Taken together, our cell culture studies identified a very potent inhibitory activity of WFA on RbTCF cell proliferation and differentiation, whereas, blockade of cell migration in RbTCFs required higher concentrations of WFA.

### WFA potently targets soluble vimentin in Tenon's capsule and downregulates the skp2-p27^kip1^ pathway in experimental glaucoma filtration surgery

**Figure 5:**
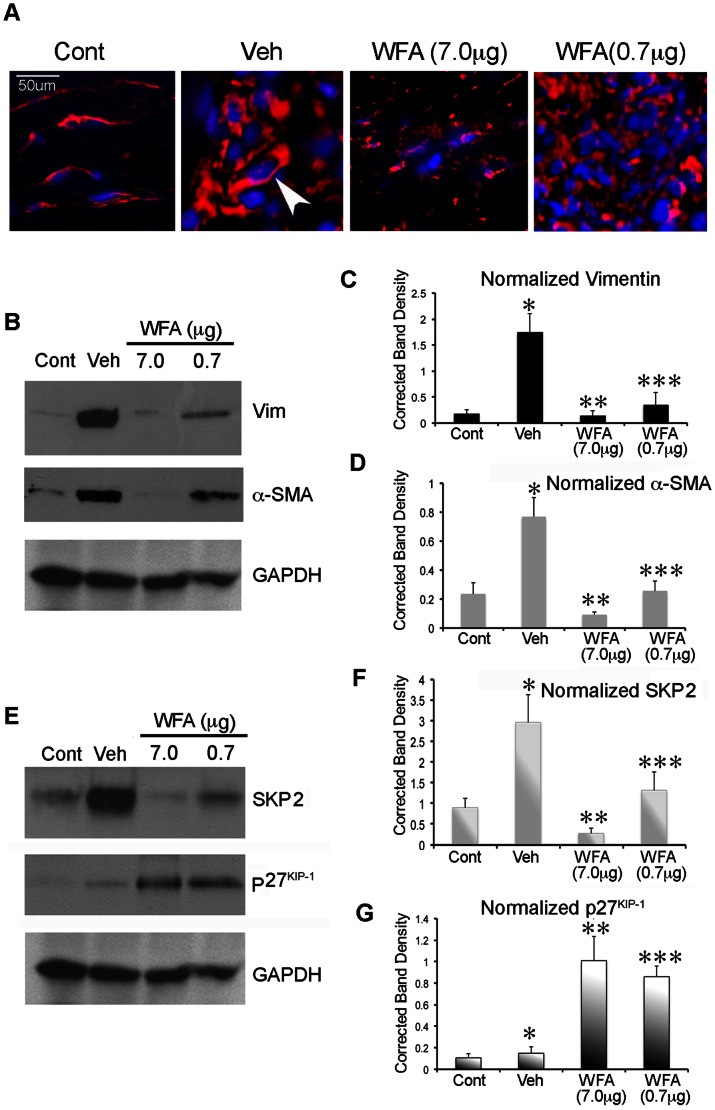
WFA downregulates soluble vimentin and affects the skp2-p27^kip1^ anti-fibrotic pathway in an experimental glaucoma filtration surgery model in rabbit. (A) Immunohistochemical analysis of vimentin expression (*red*) in Tenon's capsule tissues at surgical sites 28 days post-injury (30× magnification). Note the change in cell shape (*arrowhead*) in the injured sample (Veh) with abundant cytoplasmic vimentin staining (*red*) as compared to uninjured sample (Cont) and the downregulation and fragmentation of vimentin-IFs in the high dose WFA-treated sample. (B and E) Western blot analysis of representative tissues from Tenon's capsule tissues extracts at 28 days post-injury showing expression levels of vimentin, α-SMA, skp2 and p27^kip1^ in low-salt buffer extracts (soluble fraction). Blots were re-probed consecutively with antibodies. (C–D and F–G) Densitometric quantification of soluble vimentin (*black*), α-SMA (*grey*), skp2 (*shaded grey*) and p27^kip1^ (*shaded black*) normalized to GAPDH, using ImageJ software. Scale bars: 50 µm at 30× magnification. *P = 0.0012 Cont vs Veh; **P = 0.0005 Veh vs WFA high dose; ***P = 0.0069 Veh vs WFA low dose (*Panel C*). *P = 0.0001 Cont vs Veh; **P = 0.0001 Veh vs WFA high dose; ***P = 0.0001 Veh vs WFA low dose (*Panel D*). *P = 0.0001 Cont vs Veh; **P = 0.00005 Veh vs WFA high dose; ***P = 0.0012 Veh vs WFA low dose (*Panel F*). *P = 0.0357 Cont vs Veh; **P = 0.0005 Veh vs WFA high dose; ***P = 0.0012 Veh vs WFA low dose (*Panel G*).

Next, to validate that WFA was also effective in targeting soluble vimentin in Tenon's capsule in the GFS model of fibrosis, we exploited a localized subconjunctival method for WFA delivery as described in Methods. In Figure 5A, representative images of injured Tenon's capsule tissues, taken at high magnification (60×), revealed intense vimentin staining (*red*) localized primarily in the enlarged cytoplasm of the resident cells (*arrowheads*). This finding revealed the transition of wound fibroblasts into the myofibroblastic phenotype, as revealed in comparison to the non-injured control tissue that showed a typical spindle-like fibroblast shape with constitutive levels of vimentin-IF staining. The high dose (7 µg) WFA treatment caused potent downregulation of vimentin, resulting in peri-nuclear condensation of vimentin-IFs and their fragmentation represented as dot-like staining [5], [6]. Notably, the low dose (0.7 µg) WFA treatment did not produce any noticeable effects on cytoskeleton vimentin-IFs.

Next the pharmacodynamic effects of the two WFA doses on soluble vimentin expression were investigated by western blot analysis. Proteins extracted in low salt buffer (soluble vimentin fraction) [6] from injury-healing Tenon's capsule tissues showed increased levels of soluble vimentin in comparison to low baseline levels found in uninjured eyes (6-fold increase; P<0.0012) (Figure 5B). The 7.0 µg high WFA dose treatment targeted the injury-induced expression of vimentin causing potent reduction of soluble vimentin expression in Tenon's capsule tissues, reducing this to levels found in uninjured controls (6-fold reduction; P<0.0005). Importantly, the 0.7 µg low WFA dose also significantly reduced soluble vimentin expression (4-fold reduction; P<0.0069) in Tenon's capsule. These data afforded us next the opportunity to validate biological relationships between WFA's binding target, soluble vimentin, and biomarker expression; correlation and regression analysis on the log transformed values revealed that biomarkers skp2 and α-SMA expression levels were strongly correlated with soluble vimentin (R = 0.86604 and R = 0.86727), respectively. Furthermore, there was also a strong correlation for the inverse relationship between skp2 and p27^Kip1^ expression levels (R = −0.78; P = 0.013), revealing that WFA's downregulation of skp2 in Tenon's capsule was protective of p27^Kip1^ injury-induced downregulation. On the other hand, we found that the lower WFA dose caused increased expression of p27^Kip1^ in Tenon's capsule (5.7-fold increase; P = 0.0012) with only small further increase being observed at the high WFA dose (6.7 fold; P<0.00005). In vehicle-treated injured tissues, a 3.4-fold induction of α-SMA expression was observed (P = 0.0001), which was potently reversed in injured tissues by the high WFA dose (P<0.0001), reducing α-SMA expression levels to that found in control samples (P = 0.1257). Interestingly, the low WFA dose also reduced the injury-induced expression of α-SMA expression (P<0.0001), however, this reduction was only moderate when compared to effect of the high WFA dose. Taken together, we established from these *in vivo* experiments that WFA potently targets soluble vimentin in Tenon's capsule in a dose-dependent manner and exerts also effective control of the skp2-p27^Kip1^ pathway.

### WFA's effects on bleb survival

**Figure 6:**
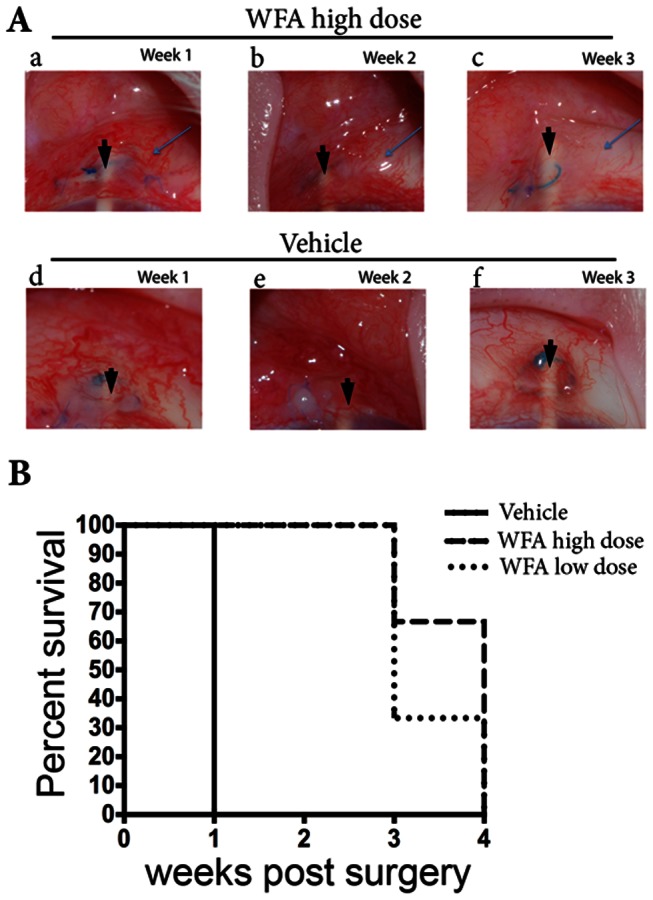
Bleb appearance in representative rabbit eyes subjected to GFS. (A) WFA treated eyes (top panels a–c; high dose) and vehicle treated eyes (bottom panels d–f) at weeks 1, 2 and 3 post-surgery. The arrows indicate the presence of the bleb and arrowheads point to the cannula. Note the increased vascularization at two weeks post surgery (e). (B) Kaplan-Meier bleb survival plot of rabbit eyes treated with vehicle (solid line), low dose WFA (dotted line) and high dose WFA (dashed line).

We also compared vehicle-treated injured eyes to those treated with WFA for assessment of the extent of bleb appearance and their survival at the times WFA injections were delivered (Figure 6A). Vehicle-treated eyes had 100% bleb survival at one week post-surgery, but by two weeks, all these blebs had failed. WFA treatment at both the high and low dose showed equivalent efficacy (100%) in extending bleb survival to two weeks (Figure 6B). The extension of bleb survival by the low WFA dose at three weeks post surgery did not reach statistical significance; however, 67% of blebs treated with high WFA dose survived. All blebs treated with both WFA doses failed at four weeks post-surgery. No adverse responses to WFA in any of the drug treated eyes were detected during the weekly clinical examinations. The intraocular pressure remained normal and no cataract or corneal irritation was observed.

### Low-dose WFA subconjunctival injection provides cytostatic drug concentrations in the anterior segment

Finally, to determine whether WFA delivery is maintained for a week in tissues surrounding the site of injection, we measured WFA levels in anterior segment tissues at one week after the last dose in a weekly multiple dosing regimen using LC-MS/MS analysis. The aqueous humor (Table 2) had the lowest WFA levels (2.52 to 4.62 ng/g), whereas almost four-times higher levels of WFA were retained in the conjunctiva (10.12 to 17 ng/g). WFA in lens tissue was below the LC-MS detection limit (0.12 ng/ml). Taken together, these data reveal that subconjunctival injection of WFA at the low dose achieves a pharmacologically relevant concentration that would maintain cytostatic activity over the treatment period of 28 days *in vivo*.

**Table 2:** WFA distribution in anterior segment tissues.

Tissue	WFA (range ng/g)	WFA (mean ng/g)	WFA (mean nM)
Aqueous humor	2.52–4.62	3.57	7.59
Cornea	4.29–7.80	6.04	12.85
Iris-ciliary body	6.07–11.37	8.72	18.55
Conjunctiva	10.12–16.99	13.55	28.82

## Discussion

The principal finding of this study is the demonstration of vimentin druggability in the GFS model of ocular fibrosis. Here, we demonstrated that WFA dose-dependently targets soluble vimentin to reduce its expression in Tenon's capsule and inhibits expression of skp2, the ubiquitin E3 ligase that governs the proteasome-mediated degradation of p27^Kip1^. This, as far as we know, is the first demonstration of vimentin targeting by localized delivery of WFA *in vivo*.

Vimentin is the principal type III IF protein expressed by fibroblasts and its expression becomes increased after an injury [28]. Moreover, vimentin-deficient fibroblasts are inept at fully engaging the myofibroblast phenotype [49]. Consequently, injured tissues of Vim KO mice show significant delay in myofibroblast expression and have an impaired wound healing phenotype [28]. Moreover, a body of literature has shown that vimentin overexpression occurs in different organs undergoing fibrosis [50], [51], [52], yet, whether vimentin is a suitable pharmacological target for surgical fibrosis had remained untested. The Tenon's capsule fibroblast cell culture model allowed us to clearly define the dose-related effects of WFA on cellular processes linked to injury repair. We identified that WFA potently targets RbTCF cell proliferation *in vitro*, demonstrating that very low concentrations of WFA (IC_50_ 25 nM) affect G_0_/G_1_ cell cycle arrest. In this respect, we found that WFA is about 2-fold more potent than MMC on inhibiting RbTCF cell proliferation [38]. The cytostatic effect of WFA confirms our previous results made in primary endothelial cells, astrocytes, and corneal fibroblasts, and also relates to the *in vivo* activity of WFA dosed at 2 mg/kg/d by i.p. injection resulting in potent inhibition of pathological angiogenesis, gliosis, and fibrosis in mice [5], [6], [7]. WFA's cell cycle-targeting activity in RbTCFs mediated through perturbation of the skp2-p27^Kip1^ pathway reflected that over 95% of cells were arrested in G_0_/G_1_ phase at 125 nM concentrations, while importantly, vimentin-IF cytoskeletal integrity and cell shape changes were unremarkable. This important result illustrates a concept that has not been as well appreciated previously since most published reports on vimentin have investigated the roles of polymeric vimentin focusing on its role in cytoskeletal-related activities [24], [25], [27], [28], [53]. Considering that soluble tetrameric vimentin is a minor fraction of the cellular protein [1], its detection by immunohistochemical staining methods is undoubtedly masked by the abundant cytoskeletal vimentin-IFs. Trypsinization of cells effectively convert polymerized vimentin to soluble vimentin; cell replating orchestrates the process of vimentin polymerization and within a few hours the vimentin-IF cytoskeletal structure is fully reestablished [54]. These newly formed vimentin-IFs have cytoskeletal structures indistinguishable from those observed in unperturbed differentiated mesenchymal cells, which underscores the dynamic nature of the vimentin cytoskeleton. Freshly plated cells rapidly extend their cytoplasm and incorporate tetrameric vimentin subunits into unit length filaments that become detectable by microscopy first as tiny dots and grow into squiggles that continue to extend laterally and become compacted into long filament structures [55] in manners mirroring polymerization of the purified protein [56]. These nascent tetrameric and short multimeric forms of vimentin are collectively considered the “soluble forms” because of their solubility in low salt buffers. Collectively, these soluble vimentin forms are highly sensitive to WFA activity in cell cultures and become incorporated into the growing filament without affecting IF polymerization, although the exact structural size of the *in vivo* target of WFA has not been characterized. We have used biotinylated WFA combined with streptavidin affinity chromatography to demonstrate that WFA binds and isolates soluble vimentin from cells [5], and this paradigm was extended also to soluble GFAP [6], and most recently, to soluble desmin [7] because of the highly conserved WFA binding site in their respective rod 2B domains. Thus, during myofibroblast differentiating WFA potently downregulated soluble vimentin and α-SMA expression in RbTCFs at concentrations under 500 nM underscoring the sensitization of soluble vimentin to TGF-ß-driven signaling events. Since TGF-ß stimulates vimentin gene expression [57] that would also increase the pool of soluble vimentin, one likely explanation for the increased sensitivity to WFA is the greater abundance of soluble vimentin. On the other hand, in our cell culture model of wound injury where less than 5% of cells engage in cell proliferation, we found that WFA's high concentration-dependent blockade of wound repair was due to inhibition of cell migration. Thus, our findings reveal that WFA exerts several dose-related biological effects in RbTCFs at non-toxic concentrations. These mechanistic findings from our cell culture studies corroborate our previous *in vivo* studies made in the alkali-induced model of corneal fibrosis that showed significant modulatory effects by WFA on skp2, p27^Kip1^ and α-SMA expression that resulted in enhanced corneal repair in mice at a non-toxic WFA dose (2 mg/kg/d; intraperitoneal injection) [7]. Consequently, we sought similarly to identify the dose-response relationship of soluble vimentin targeting by localized WFA delivery in the clinically relevant rabbit model of GFS.

The design strategy to target soluble vimentin in rabbit eyes was predicated on the binding mode of WFA to tetrameric vimentin based on our prior molecular modeling studies [5]. WFA binds to a highly conserved rod 2B region of the vimentin tetramer, where a covalent bond occurs between the epoxide group of WFA and the cysteine residue of the protein. We rationalize that native tetrameric vimentin is bound covalently by WFA through two discrete sequential steps. WFA binds initially with key amino acids in the binding pocket through hydrophobic interactions made with the polycyclic hydrocarbon chain backbone of the steroidal lactone. Additionally, hydrogen bonding between glutamine and aspartic acid with the A-ring groups of WFA are critical for activity as their modification alleviates biological effects of the compound [5]. Once WFA is oriented in the correct position via these reversible interactions, a second irreversible binding event occurs when the properly aligned nucleophilic cysteine of vimentin attacks the C6-ß-epoxide of WFA. The remarkable stereospecific requirement for attack on the C6-ß-epoxide predicts the correct binding pose of WFA in the pre-alkylation complex, because the close structural congener, 12-deoxowithastramonolide (12D-WS) that also contains a reactive epoxide ring that is oriented instead in the alpha-position, does not afford a covalent binding with tetrameric vimentin. Consequentially, 12D-WS was found not to possess the specific biological activities of WFA in *in vitro* and *in vivo* assays [5], [16]. Notably, prior results have also shown that WFA concentrations even 10-fold higher than that (∼3 µM) capable of exerting significant vimentin-IF structural collapse and causing apoptosis in cell cultures did not *per se* disrupt the formation of vimentin-IFs in cell-free *in vitro* vimentin polymerization assays [5], [17]. Hence, WFA's perturbation of vimentin-IFs in cell cultures that occur at non-toxic submicromolar concentrations likely engage complex intracellular regulatory mechanisms (e.g. phosphorylation or protein-protein interactions with chaperones), which are known to govern the structural dynamics of this protein class [53], [58], [59], [60]. We believe that such interactions with diverse co-associated proteins constitute a higher order of regulatory control over vimentin's filamentous dynamic behavior in cells. In fact, known cytoskeleton vimentin-interacting proteins were isolated using a WFA-biotin analog, which revealed that ß-actin and ß-tubulin [5] as well as myosin (our unpublished data) co-isolate with affinity tagged vimentin. These findings illustrate that the vimentin-IF protein-interactome is both large and complex, and that signaling events that alter cellular states can engage the vimentin-IF cytoskeleton and affect its organization. For instance, vimentin-IFs can dissemble and assemble their cytoskeletal structures at lamellipodia [24] and filopodia [61] enabling these mobile stress fiber scaffolds to rapidly respond locally to biological cues. This suggests that the druggable soluble forms of vimentin will be governed by their local abundance and temporal regulation, which could be the critical pharmacodynamic aspects of this class of IF protein target. In this respect, studies investigating modalities that affect the mechanical properties of cells have shown that cell softening induced by myosin perturbation promotes the availability of soluble vimentin by depolymerizing polymeric vimentin-IFs, thereby exposing the vimentin cysteine group to labeling by cysteine-reactive chemical probes. However, in unperturbed stiff-cells due to low levels of soluble vimentin the cysteine-reactive probe did not label cells due to the predominantly polymeric forms of vimentin-IFs [62]. Taken together, the idea advanced from our first part of this study that helped define WFA's dose-related targeting of soluble vimentin in RbTCFs afforded us a rationalized mechanistic insight to probe the vulnerability of the vimentin-skp2-p27^kip−1^ axis to WFA perturbation *in vivo*.

In this second part of the study, we ascertained that uninjured rabbit eyes expressed stable low baseline levels of soluble vimentin in Tenon's capsule tissues. Hence, rabbit eyes subjected to GFS that showed sustained high levels of soluble vimentin in Tenon's capsule post injury afforded us a model to investigate soluble vimentin targeting in response to localized WFA treatment. Our findings showed a dose-dependent reduction of soluble vimentin after WFA treatment, and identified a pattern of p27^kip1^ expression that was similar to that in RbTCF cell cultures showing maximal expression at low nanomolar concentrations. Indeed, tissue concentrations of WFA achieved by localized delivery in the anterior segment of rabbit eyes corresponded to an *in vitro* concentration that caused G_0_/G_1_ cell cycle phase growth arrest in cultured RbTCFs. Since reduction of skp2 levels was also achieved by the low WFA dose *in vivo*, our data suggests that the skp2-p27^kip1^ cell cycle pathway [43] was attenuated at this dose. However, as the high WFA dose caused even more prominent downregulation of skp2 and α-SMA, this suggested that other mechanisms beyond inhibition of cell proliferation were involved in mediating WFA's high dose anti-fibrotic activity. In this respect, skp2 has recently been shown to also regulate cell migration, which occurs in a p27^kip1^-independent manner [63]. Skp2-deficient embryonic fibroblasts were shown to be defective in cell migration and this inhibition was overcome by re-introduction of recombinant skp2 that lacked its nuclear localization sequence [64]. Thus, cytoplasmic-localized skp2 was found not to interfere with nuclear p27^kip1^ expression or regulate cell proliferation. Direct evidence that skp2 activation is linked to fibrosis was shown in a kidney fibrosis model, where increased levels of skp2, vimentin and α-SMA were reported in fibrotic tissues [65]. In skp2 deficiency, injured mice were protected from fibrosis, which was correlated with downregulated expression of both α-SMA and vimentin, and increased expression of p27^kip1^ [65]. Given that pharmacologically we have illustrated a similar attenuation of fibrotic biomarker expression profile in injured Tenon's capsule with WFA (reduced vimentin, skp2 and α-SMA expression and increased p27^kip1^ expression) in GFS, we believe this anti-fibrotic molecular signature is an attractive mechanism worthy of pursuit in glaucoma surgery because it is also pharmacologically tractable.

Finally, our clinical observations made during the 4-week investigation using the rabbit GFS model provided some interesting preliminary results. Firstly, there was no incidence of WFA-induced adverse effects, such as increased intraocular pressure, cataracts, or corneal irritation in any of the rabbits during drug treatment, suggesting that both doses of WFA were well tolerated. Because WFA-treatment at both doses showed equivalent bleb survival up to 2 weeks post-surgery, we wondered if this was due to WFA's anti-proliferative mechanism because it is known that cell proliferation is most prominent at the early (1 to 2 week) stages of injury repair in GFS [66]. We rationalize that WFA's attenuation of cell proliferation would be readily achieved at the lower dose. As there is indication that bleb survival was extended further by the higher WFA dose, it is possible that at later stages other mechanisms of the fibrotic repair process become relevant that were not vulnerable to the low WFA dose. For instance, during the 2 to 3 week period post-injury, myofibroblasts become prominently elaborated in GFS [67]. During injury healing, such contractile cells utilize cytoplasmic vimentin-IFs as mechanical force-bearing structures to orchestrate coherent cytoskeleton-mediated cell movement [28]. We rationalize that the 10-fold higher WFA dose would likely interfere with the cytoskeletal cell migratory machinery as we found only the high WFA dose affected vimentin-IFs *in vivo*. In regards to this mechanism, it was recently reported that polymeric vimentin IFs contribute significantly to focal adhesion growth, endoplasm spreading and cell motility, which are cellular mechanisms involved in cell migration that were shown to be inhibited by WFA [17], [18] at concentrations considerably higher than WFA's anti-proliferative cytostatic dose [6], [7], [14]. Hence, we speculate that attenuation of cell migration and cell contractile activity [68] of myofibroblasts by the higher WFA dose may be linked to the longer extension of bleb survival in GFS. This prospective idea obviously requires further detailed investigation to define the temporal and dose-related pharmacodynamic effects of WFA on key molecular mediators of cell proliferation and migration that are important for bleb survival in this GFS model.

This pilot *in vivo* study also has some limitations. First, solid evidence to support that the higher WFA dose increases bleb survival times will require greater numbers of rabbits to confirm this pilot study. Second, the target and biomarker measurements we made have used tissues that were obtained at terminal end points and not at weekly stages when bleb survival was recorded. This suggests that interpretations made on target druggability and biomarker response relate only to states of the prevailing pathophysiology of Tenon's capsule at 28 days post injury, and not directly to bleb survival. Indeed, experiments that assess target and biomarker expression at each survival time point will need to be performed. This will allow us to validate survival outcome with biomarker protein expression characteristics that define WFA's mechanisms more accurately. Moreover, the retention of higher concentrations of WFA in conjunctival tissues is consistent with the hydrophobic nature of WFA and its affinity for lipid membranes. The absence of detectable levels of WFA in lens is consistent with the hydrophilic nature of abundant lens proteins and low affinity of WFA for this richly proteinaceous tissue. The low levels of WFA in aqueous humor are probably reflective of the free drug concentrations in surrounding tissues. Thus, one may anticipate that the highest therapeutic levels of WFA are achieved and maintained in the conjunctiva and surrounding tissues including Tenon's capsule. However, low but significant WFA levels were detectable in off target tissues also. As such, ophthalmic toxicological studies have yet to be undertaken on WFA and any potential side effects after prolonged treatment remain to be determined. Our future studies will investigate target-related and off-target effects of this inhibitor. As such, all these deficiencies of the current investigation will require large-scale rabbit studies that were beyond the scope of our pilot investigation. We believe the data nevertheless is very informative for designing such future studies as we have demonstrated for the first time vimentin druggability using a localized delivery paradigm in rabbits. In conclusion, our proof-of-concept investigation has provided pivotal evidence that soluble vimentin in Tenon's capsule is druggable and this mechanism potently engages the skp2-p27^kip1^ pathway. Given that WFA has witnessed prolific interest lately from diverse clinical areas of medicine for its vimentin-targeting activity [6], [8], [9], [10], [69], [70], the use of localized delivery methods for WFA could offer additional insights into disease mechanisms sensitive to vimentin chemical perturbation. Our findings illuminate, additionally, the potential of developing localized delivery methods to target other type III IFs (GFAP, desmin, peripherin) where the overexpression of these homologous type III IFs has been related to pathological processes such as reactive gliosis 6,71,72 and related IF-mediated disorders [7], [73], [74].
